# An Easy-to-Use Procedure for the Measurement of Total Phenolic Compounds in Olive Fruit

**DOI:** 10.3390/antiox10111656

**Published:** 2021-10-21

**Authors:** Pilar Luaces, Mar Pascual, Ana G. Pérez, Carlos Sanz

**Affiliations:** Department of Biochemistry and Molecular Biology of Plant Products, Instituto de la Grasa, Spanish National Research Council (CSIC), 41013 Seville, Spain; pluaces@ig.csic.es (P.L.); marpa@ig.csic.es (M.P.); agracia@ig.csic.es (A.G.P.)

**Keywords:** *Olea europaea* L., phenolic compounds, olive fruit, virgin olive oil, quality

## Abstract

Virgin olive oil (VOO) is one of the most emblematic products of the Mediterranean diet. Its content in phenolic compounds is strongly associated with the antioxidant and health-promoting properties of this diet. VOO’s phenolic profile is determined mainly by the phenolic compounds present in the olive fruit, so knowing their content allows for a fairly precise estimate of the antioxidant and functional properties of the corresponding oil. In this sense, a convenient, green, and sensitive spectrophotometric method was developed for the quantitative determination of total phenolic compounds in olive fruits. The method is based on an easy-to-use extraction procedure of olive fruit phenolics using dimethyl sulfoxide and quantification with the Folin–Ciocalteu reagent. Oleuropein proved to be a suitable reference compound for quantification, displaying a good linear response (r = 0.9996) over the concentration range of 0.58–6.48 mg/mL, with a variation coefficient of 0.42% and limits of detection and quantification of 0.0492 and 0.1490 mg/mL, respectively. The method was validated using a wide array of fruit samples representative of the *Olea europaea* L. genetic diversity. The results obtained with this spectrophotometric method, expressed as mg/mL of oleuropein, showed a good correlation with those obtained with the fruit samples analyzed by high performance liquid chromatography, with an *r* value of 0.9930 and a slope value of 1.022, confirming its reliability. Thus, this method can become a very useful simple tool to estimate the total phenolic content of olive fruits, especially when working with numerous samples such as in olive breeding programs or in commercial olive production, in which it is especially useful to know the phenolic state of the fruit and thus determine the optimal harvest date or the most appropriate agronomic treatment to increase the functional properties of the olive fruit and the olive oil.

## 1. Introduction

The phenolics of virgin olive oil (VOO) have attracted special attention in recent years for their proven properties that promote human health, particularly regarding cardiovascular diseases, inflammation, or cancer [1,2]. Thus, a health claim on the evidence of the protective effect of VOO phenolics for cardiovascular diseases was approved by the European Food Safety Authority [3], which applies to those oils that contain at least 250 ppm of hydroxytyrosol and derivatives. Furthermore, phenolic compounds also play an important role in the sensory profile of VOO, as the bitter and pungent sensory notes that characterize VOO have been associated with various phenolic compounds [4,5,6,7]. The phenolic fraction of VOO is made up of an array of compounds belonging to different chemical classes such as secoiridoids, lignans, flavonoids, a series of alcohols, and simple phenolic acids, with the main phenolic derivatives of oil being the secoiridoids. The presence of secoiridoids in VOO is related to the content of phenolic glycosides with the secoiridoid structure present in the olive fruit, whose main representatives are oleuropein, ligstroside, and demethyloleuropein, and the activity of the hydrolytic and oxidative enzymes that act on these glycosides during the oil extraction process [8,9,10]. In fact, we have recently found a very significant correlation between the main phenolic groups in olive fruit and VOO, which suggests that the phenolic composition of the fruit is one of the main factors responsible for the phenolic content of VOO [11]. 

Dimethyl sulfoxide (DMSO) is a non-toxic aprotic and amphiphilic solvent, which is soluble in water and organic solvents and capable of dissolving a large number of lipophilic compounds. Its antioxidant properties make DMSO a very suitable extraction solvent to avoid the degradation of phenolic compounds. Thus, Sricharoen et al. [12] observed that DMSO-extracted chili phenolics had the highest in vitro antioxidant activity value with any of the most commonly used antioxidant assays compared to other common solvents. In this regard, we developed a fast, reliable, and flexible procedure for the extraction of the main phenolic compounds in olive fruit based on the use of DMSO as an extractant agent, which through subsequent high performance liquid chromatography (HPLC) analysis allows us to know the phenolic profile of this fruit [8]. This extraction procedure proved to be very suitable for the conservation of olive phenolic extracts for long periods of time both in cold and at room temperature [13].

Many analytical procedures have been developed for the quantification of total phenolic content in foods [14]; however, although separative methods, such as HPLC with UV-vis detection, may be powerful techniques for the identification and quantification of phenolic compounds in complex samples, their use to estimate the total phenolic content may be inaccurate [15]. Furthermore, the separative techniques are solvent- and time-consuming, expensive, and often not suitable for routine determinations. For this reason, the Folin–Ciocalteu (FC) assay [16,17] has been proposed as a standardized method for use in routine quality control and measurement of antioxidant capacity for many food products. In fact, the determination of total phenolic content using the FC assay is the method most frequently applied in foods for total quantification of phenolic compounds [18].

Phenolic compounds are currently being used as quality markers for VOO due to their organoleptic and health promoting properties. As a consequence, the phenolic components of olive fruit are now being used as quality traits in olive breeding programs or for the assessment of agronomic practices on the fruit phenolic status [19,20,21]. Due to the long juvenile phase and unproductive period of olive, a routine selection process in olive breeding programs can take up to 20 years. Among the methodological advances carried out for the establishment of early selection criteria, thus reducing the time and effort for advancing the selection process, we are currently involved in the development of new genomic tools such as genome-wide association studies (GWAS), which narrows the genomic regions to search for potential candidate genes and causal polymorphisms involved in the synthesis of olive phenolic compounds. This requires the evaluation of a considerable number of both olive genomes and phenolic contents. It has therefore become necessary to have a simple and flexible procedure for the extraction and measurement of the content of phenolic compounds from numerous fruit samples at the same time. This system would also be very useful during olive production since it would allow for rapid decision making to estimate the harvest time or to modify the cultivation conditions in order to increase the phenolic content in the fruit and, consequently, increase the functional quality of the corresponding oil. Accordingly, the objective of this work was to develop and validate a reliable analytical procedure for the quantification of total phenolic compounds in olive fruit samples, taking advantage of the suitability of both DMSO as an extractant and the commonly used FC reagent for the easy measurement of total phenolic compounds.

## 2. Materials and Methods

### 2.1. Reagents 

All reagents were standard analytical grade. DMSO, FC reagent, sodium carbonate, and phosphoric acid were purchased from Sigma-Aldrich (Sigma-Aldrich Co., St. Louis, MO, USA). HPLC-grade methanol and acetonitrile were from Panreac (Panreac Applichem, Barcelona, Spain). Solutions were prepared using ultrapure water produced by Milli-Q water purification system (Millipore, MA, USA).

### 2.2. Plant Material

For method validation, phenolic extracts were obtained from fruits along development and ripening of different olive cultivars selected on the basis that they produce oils with very different profiles and contents of phenolic compounds [11]. Olive trees (*Olea europaea* L.) of seven cultivars and three genotypes of the crossing of cultivars Picual x Arbequina were used: olive cultivars with high levels of phenolic compounds, Dokkar, Menya, and Piñonera; middle levels, Picual, Arbequina, UCI-6, and UCI-7; and low levels of phenolic compounds, Abou kanani, Shengeh, and UCI-5. 

Trees, two per accession, were grown in the same agroclimatic conditions at the experimental orchards of the Instituto de la Grasa in Seville. They had a 6x5 m spacing plantation frame and an irrigation regime with drip fertigation from the moment of flowering until the complete ripening of the fruit. Fruit harvest was carried out by hand throughout the fruit development process (stages D-I, D-II, and D-III) and ripening (stages M-I, M-II, and M-III). The age of the olive fruit was established in weeks after flowering (WAF) during development, while during ripening, it was related to the maturation index (MI) determined on the basis of the color of the skin and the mesocarp of the olive fruit [22]:

D-I, olive fruits after endocarpal hardening, 12 WAF;

D-II, olive fruits with 16 WAF;

D-III, olive fruits with 20 WAF;

M-I, olive fruits with green-yellowish epidermis (MI = 1);

M-II, turning olive fruits, 50% color (MI = 2.5);

M-III, olive fruits with dark purple epidermis and white mesocarp (MI = 4).

### 2.3. Extraction of Fruit Phenolic Compounds

Phenolic compounds were extracted from olive fruits according to Fernández et al. [13]. Thin longitudinal pieces of mesocarp tissue were cut from 20-40 olive fruits and submerged in DMSO (6 mL/g of fruit). The tissue was then homogenized with Ultraturrax (1 min × 10,000 rpm) and subsequently centrifuged in a minifuge (2 min × 600× *g* ) to obtain the phenolics extract, or a passive extraction of the phenolic compounds was carried out in a refrigerator (4 °C) for 72 h. In the latter, the extraction process is assisted with a 10 min ultrasound bath (Branson 3510, Branson Ultrasonics Corporation, Danbury, CT, USA) before and after the time in the refrigerator. Syringic acid (24 µg/mL DMSO) was used as internal standard. Phenolic extracts were then filtered through 0.45 μm nylon filters and kept at −20 °C until HPLC analysis. Fruit phenolic extracts were carried out in duplicate.

### 2.4. Folin–Ciocalteu Test

Total phenolic content was determined with the FC reagent based on a procedure described by Singleton and Rossi [16]. For sample preparation, an aliquot of the phenolics extract (typically 20 µL), 3 mL of deionized water, and 250 µL of FC reagent were placed in a 10 mL test tube, shaken, and allowed to stand protected from light for eight minutes. Then, 750 µL of a 7.5% sodium carbonate solution was added and made up to a volume of 5 mL with distilled water. The solution was homogenized manually and kept in the dark at room temperature. The absorbance of the mixture was measured at 765 nm after 2 h (±5 min) against a reagent blank with a Beckman-Coulter DU-800 spectrophotometer, equipped with tungsten (visible) and deuterium (ultraviolet) lamps (Beckman Coulter Inc., Brea, CA, USA). All the experiments were carried out in triplicate.

For the implementation of the FC test, the previous construction of calibration curves was necessary for different compounds representative of the main families of phenolic compounds present in the olive fruit, oleuropein (secoirdoids), rutin (flavones), and cyanidin-rutinoside (anthocyanins), as well as gallic acid since it is the most common reference compound in FC tests.

### 2.5. HPLC Analysis of Fruit Phenolic Compounds

Phenolic compounds were analyzed in a Beckman Coulter liquid chromatographic system (Beckman Coulter Inc., Brea, CA, USA) equipped with a diode array detector (System Gold 168) and a Superspher RP 18 column (4.6 mm i.d. × 250 mm, particle size 4 µm: Dr. Maisch GmbH, Ammerbuch, Germany) under the system conditions previously described [11]. Elution was performed at a flow rate of 1.0 mL/min and a temperature of 35 °C, using phosphoric acid 5 g/L (solvent A) and methanol/acetonitrile (1:1, *v:v*) (solvent B) as the mobile phases. Phenolics were monitored at three different wavelengths, 280, 335, and 517 nm, and quantified taking into account the internal standard and response factors for each of them. The identity of phenolic compounds was established based on their UV-vis spectra and available standards and confirmed by HPLC/ESI-qTOF-HRMS analysis [11]. The phenolic compounds were grouped into different groups according to their nature as follows: hydroxytyrosol (HTy) derivatives (oleuropein, demethyloleuropein, oleuropein aglycone, HTy-glucoside, verbascoside), tyrosol (Ty) derivatives (ligstroside, demethylligstroside, ligstroside aglycone, Ty-glucoside), flavones (rutin, luteolin-glucoside, apigenin-glucoside), and anthocyanins (cyanidin-rutinoside, cyanidin-glucoside).

### 2.6. Statistical Analysis 

Statistical analysis was performed using Excel 2016 and STATISTICA (Statsoft Inc., Tulsa, OK, USA). A *p* value less than 0.05 was considered to be statistically significant.

## 3. Results and Discussion

Procedures that do not require sophisticated and expensive facilities and equipment are necessary for a fast and reliable assessment of the total phenolic contents in olive fruit samples to help in olive breeding programs or for the assessment of agronomic practices on the fruit phenolic contents. To this end, the convenient phenolic extraction procedure previously developed using DMSO [13] in combination with the spectrophotometric quantification using the FC reagent has been explored. For this purpose, oleuropein has been used as a reference compound to express the content of phenolics as oleuropein equivalents since we have previously found that on average this compound represents close to 80% of the phenolic compounds in the olive fruit. In addition to oleuropein, ligstroside and demethyloleuropein, the major phenolic glucosides in olive fruit, and others such as verbascoside and the flavonoids rutin, luteolin, and apigenin glucosides are also present in significant amounts [11]. Furthermore, anthocyanins are synthetized during fruit ripening and constitute a major class of compounds within the olive fruit phenolics.

**Figure 1:**
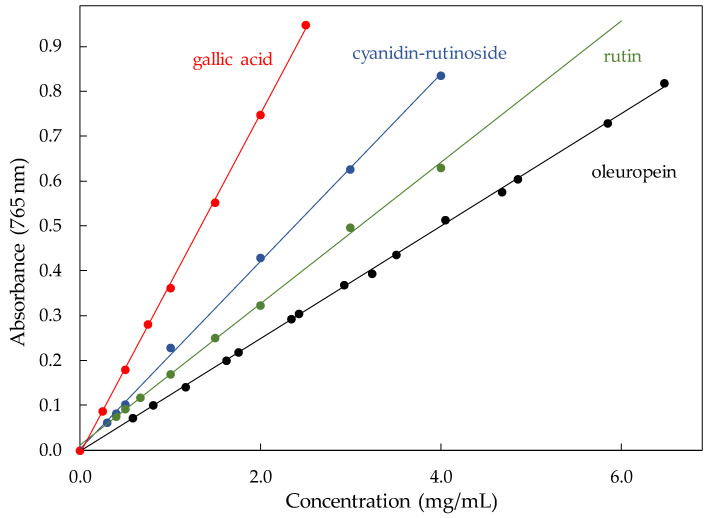
Calibration curves for oleuropein, rutin, cyanidin-glucoside, and gallic acid. Data on linear regression analysis for each compound are shown in Table 1.

First, a study of the reaction of oleuropein in DMSO with the FC reagent has been carried out taking into account the content ranges previously found for phenolic compounds in an olive collection representative of the olive genetic diversity [11,23] and the absorbance range in which the Lambert–Beer law is fulfilled. To this end, two different calibration curves for oleuropein have been obtained and combined. Figure 1 showed the relationship between oleuropein concentrations and the corresponding absorbance at 765 nm after reaction with the FC reagent. The reaction displayed strong linearity as shown by least square analyses (Table 1), with a determination coefficient (R^2^ = 0.9992) higher than 0.995, which according the Eurachem guide [24] indicates that the analytical response was linear. The good regression coefficient (*r* = 0.9996) throughout the range and a relative standard deviation of the regression (RSD_b_ = 0.42%) lower than 5% confirm the linearity. Additionally, calibration curves for the most relevant compounds within the other major groups in the olive fruit phenolics were obtained for comparison: rutin for flavones and cyanidin-rutinoside for anthocyanins. Additionally, a calibration curve for gallic acid was produced, since it is the reference compound most commonly used in the measurements of total phenols with the FC reagent (Figure 1 and Table 1). Reaction with the FC reagent for all of them presents good linearity, with determination coefficients higher than 0.995 [24]. Regression coefficients were higher than 0.999, except for rutin (*r* = 0.9977), and responses were considered linear as their RSD_b_ values were lower than 5%. These compounds present different levels of response compared to gallic acid whether expressed as mg/mL or millimolar concentration (Table 1). When expressed in millimolar concentration, it is observed that cyanidin-rutinoside reacts with the FC reagent to a greater degree (1.95 times) than gallic acid, followed by rutin (1.38 times) and, finally, oleuropein, which is practically equivalent to the reaction of the FC reagent with the gallic acid (1.05 times).

The matrix effect was examined by adding a phenolic extract of cultivar Arbequina (D-I) olive fruits (20 µL) to oleuropein solutions whose concentrations were within the range used for the calibration curve. The resulting line also presented a good linearity (*r* = 0.9992) and an RSD_b_ (0.76%) less than 5%. The slope of the line was calculated (0.1254), which coincides with that obtained for oleuropein (Table 1) with a 0.13% variation. Likewise, it was observed that the x-intercept corresponds to a theoretical concentration value of phenolic compounds of 2.047 mg/mL, which is quite close to that obtained after HPLC analysis (1.979 mg/mL). Accordingly, the matrix effect could be considered practically negligible throughout the entire concentration range under study.

The working range is limited by the values of the detection or quantification limits at the lower end, while the upper end is subject to the linear response by compliance with the Lambert–Beer law, not exceeding absorbance values of 1 at 765 nm. The limits of detection (LOD) and quantification (LOQ) were calculated from calibration curve data as 3 × *S*_a_/b for LOD, and as 10 × *S*_a_/b for LOQ, where *S*_a_ is the standard deviation of the y-intercept, and b is the slope of the calibration curve [25]. For oleuropein, an LOD of 0.0492 mg/mL and an LOQ of 0.1490 mg/mL were found (Table 1). Only the LOD and LOQ calculated for cyanidin-rutinoside were slightly lower than those found for oleuropein among the four compounds under study. 

The accuracy of the method was studied by assessing its components, precision and trueness. The former was studied in terms of repeatability and reproducibility. The repeatability of the developed method was evaluated from twenty consecutive determinations under repeatability conditions (the same sample, the same operator, and the same equipment at the same time) of two samples of fruit phenols corresponding to different cultivars and maturation stages (Picual M-I and Arbequina D-III) that had very different contents of phenolic compounds, being representative of the lower and upper part of the calibration curve. Meanwhile, reproducibility was also evaluated by analyzing both olive phenolic samples in two different spectrophotometers in different days with different operators. The precision associated with the repeatability and reproducibility conditions can be quantified using the repeatability (*r*) and reproducibility (*R*) limits. These values describe the maximum difference between two results obtained under specific conditions that can be attributed to the precision of the method and is calculated as 2.8 × *S*_r_ for *r* and 2.8 × *S*_R_ for *R* according to Rao [26], *S*_r_ and *S*_R_ being the repeatability and reproducibility standard deviations, respectively. Thus, in terms of absorbance at 765 nm, *r* and *R* values of 0.0168 and 0.0205, respectively, have been found for the experiments with Picual M-I, whereas in the case of the Arbequina D-III sample, the values found were 0.0167 and 0.0628, respectively (Table 2). In addition, the RSD values calculated for *r* and *R* were less than 5% in both cases, confirming the precision of the method throughout the working range.

Trueness was assessed through recovery studies, understood as the proportion of oleuropein added to a representative olive phenolic extract (Arbequina D-I) that is measured [27]. For this, seven additions were made in the concentration range of 0.315–3.150 mg/mL. The determination coefficient obtained for the spiked curve produced was higher than 0.995 (R^2^ = 0.9984). Moreover, linearity was confirmed through a calculated relative standard deviation of the regression lower than 5%. The recovery percentage obtained was 99.4%. Trueness was then assessed by comparing the *t*-test of the apparent mean concentration with 100% [28]. The recovery results were corroborated given that the calculated *t*-value (*t_exp_*) was 0.82, less than the tabulated *t*-value found for two sides for α = 0.05 and n − 1 degrees of freedom.

**Figure 2:**
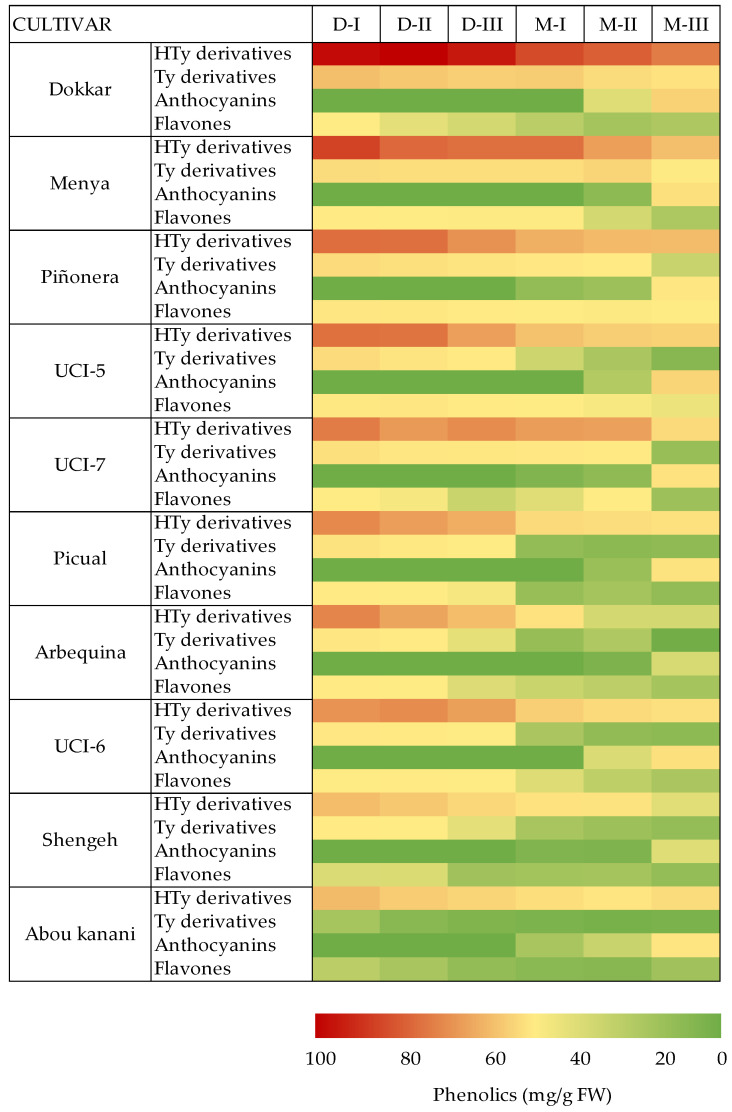
Heat map of the content of phenolic compounds (mg/g FW) in olive fruits from different cultivars along development (D-I, D-II, D-III) and ripening (M-I, M-II, M-III).

**Figure 3:**
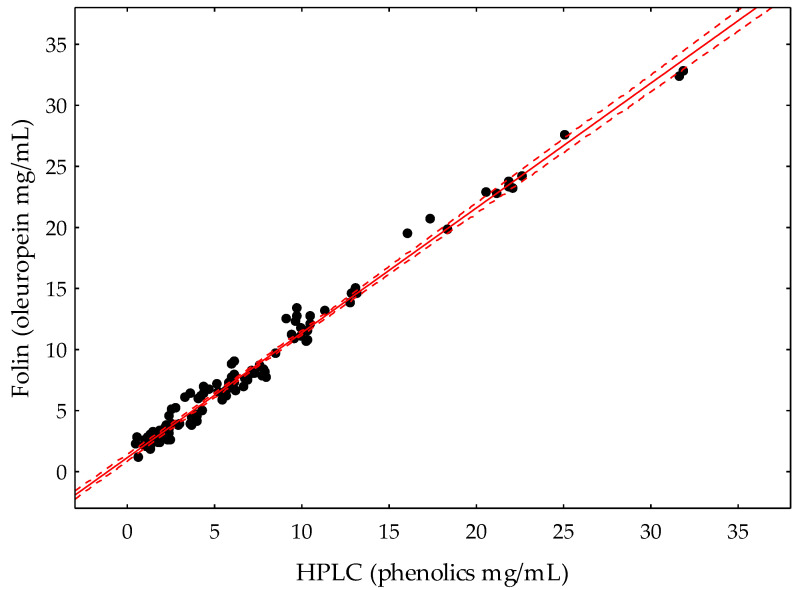
Comparison of phenolic contents in olive fruit samples analyzed by the new Folin–Ciocalteau-based method expressed as oleuropein and by HPLC, expressed as the sum of the main phenolics in the olive fruit described in Material and Methods. Dashed lines correspond to the 99% confidence interval for the regression line.

The procedure was validated by comparison with the results of the HPLC analysis performed on fruit extracts of ten different olive cultivars throughout the development and ripening of the fruit, from 12 WAF (after hardening of the endocarp) to full colored fruits (24–34 WAF). This selection of cultivars covers the genetic diversity of the olive species in relation to the profiles and contents of phenolic compounds in the olive fruits and their corresponding oils [11]. It includes cultivars characterized by having very high levels of phenolic compounds in the fruits, such as Dokkar and Menya, to others with very low levels, such as Shengeh or Abou kanani, which gives rise to a range of very different contents of phenolics. Figure 2 shows a summary of the phenolic content data of the main classes of phenolic compounds found in the olive fruit of these cultivars throughout development and ripening. Thus, the phenolic contents of olive fruit extracts obtained along fruit development and ripening were determined by the proposed FC method and compared to the HPLC data. The total phenolic content of the various olive fruit samples measured by HPLC ranged from 0.52 (Shengeh M-III) to 22.64 mg/mL (Dokkar D-I). The proposed FC method showed good linear correlation with the HPLC data (Figure 3), displaying a regression coefficient above 0.99 according to the least squares method (*r* = 0.9930). The slope of the curve was 1.022, within the calculated confidence interval at 95% (0.9999–1.0445). Accordingly, the proposed method is valid for estimating the phenolic content of olive fruit. However, higher concentrations were systematically found with the proposed method compared to those obtained by HPLC analysis. This outcome could be explained considering that the FC method estimates the content of reducing compounds, phenolic and non-phenolic, whereas the HPLC analysis only measures the content of the major phenolics in the olive fruit. Thus, assuming that most of these reducing compounds correspond to an array of phenolic compounds found in low concentration in olives, the proposed method based on the reaction with the FC reagent seems more suitable for the quantification of total olive fruit phenolics than the HPLC analysis. In this sense, Reboredo-Rodríguez et al. [29] also observed a good correlation between the total phenolic content determined by HPLC and by an FC-based method for the determination of phenolic compounds in VOO obtained after liquid-liquid extraction. In the case of VOO, the prior separation of phenolics is necessary since the FC test cannot be carried out in the presence of the triglyceride matrix, with liquid-liquid extraction and solid phase extraction being the main fractionation systems [30,31]. However, recently, different spectrophotometric approaches for the quantification of total phenolic compounds in unfractionated VOO have been published [32,33]. 

The FC reagent is considered nonspecific by many authors since it can be reduced by non-phenolic compounds [34], so it is usually necessary to improve the specificity of the FC assay through the physical or chemical elimination of interferences or the subtraction of its contribution to the reaction with the FC reagent [35]. However, according to the high degree of correlation found between the FC and HPLC methods used to evaluate the total phenolics in the olive fruit, it can be concluded that, in general, there are hardly any compounds that can interfere with the FC reaction in the phenolic extracts obtained with DMSO. This is in accordance with the results obtained in the evaluation of the matrix effect described above.

## 4. Conclusions

The proposed method developed combines the convenience of extraction with DMSO and the ease of measurement with the FC reagent by spectrophotometry, making it a convenient, green, and sensitive method for the quantitative determination of total phenolic compounds in olive fruit. The total phenol content can be expressed either as the traditional gallic acid equivalent concentration or as oleuropein concentration, which gives a better idea of the actual content of total phenolic compounds in the olive fruit. The use of DMSO as an extractant agent allows adequate flexibility for field work since it allows the fruit sample to be soaked into the DMSO directly in the field and be transported to the laboratory protected by the same solvent until the moment of analysis. Thus, the sample may be analyzed after 72 h of cold passive extraction, or at any time by using a homogenizer and subsequent centrifugation. Regarding the applicability of the developed method, the analysis of the phenolic compounds of the olive fruit can be very useful in olive breeding programs, taking into account the relationship that exists between the phenolic fraction of the olive fruit and that of VOO, responsible for its sensory and health-promoting properties, thus allowing the identification of the olive genotypes that produce oils with better functional quality. It can also be very useful as rapid decision-making procedure in current commercial cultivation since it would allow modifying the conditions of agricultural practices to increase the oil phenolic content or establish the appropriate harvest date that allows for obtaining a high oil yield along with high levels of health-promoting components.

## Figures and Tables

**Table 1:** Linear regression analysis for the reactions of oleuropein, rutin, cyanidin-glucoside, and gallic acid with the Folin–Ciocalteau reagent expressed as mg/mL or in millimolar concentration.

	Oleuropein	Rutin	Cyanidin-Rutinoside	Gallic Acid
Regression coefficient (*r*)	0.9996	0.9977	0.9992	0.9998
Determination coefficient (R^2^)	0.9992	0.9954	0.9985	0.9995
RSD_b_	0.4231	1.1464	0.7469	0.4873
Intercept (a)	−0.0031	0.0266	0.0032	−0.0102
*S*_a_	0.0019	0.0046	0.0032	0.0026
Concentration as mg/mL				
Slope (b)	0.1256	0.1457	0.2116	0.3802
*S*_b_	0.0005	0.0017	0.0016	0.0019
LOD	0.0827	0.1540	0.0763	0.1203
LOQ	0.2757	0.5133	0.2544	0.4011
Concentration as mM				
Slope (b)	0.0679	0.0890	0.1260	0.0647
*S*_b_	0.0003	0.0010	0.0009	0.0003
LOD	0.0447	0.0940	0.0454	0.0205
LOQ	0.1490	0.3133	0.1515	0.0682

**Table 2:** Precision study data using fruit phenolics extracts from two different olive cultivars and developmental stages (Picual M-I and Arbequina D-IIII).

	Picual M-I	Arbequina D-IIII
*r* (Abs. 765 nm)	0.0168	0.0167
RSD_r_ (%)	1.5751	0.9477
*R* (Abs. 765 nm)	0.0205	0.0628
RSD_R_ (%)	2.0630	3.8260

## Data Availability

Data is contained within the article.
